# FINDING THE MINDS OF OUR ELDERS: TESTING THE MINORITY STRESS AND COGNITION MODEL WITH INDIGENOUS OLDER ADULTS

**DOI:** 10.1093/geroni/igac059.1687

**Published:** 2022-12-20

**Authors:** Cliff Whetung

**Affiliations:** NYU Silver School of Social Work, New York, New York, United States

## Abstract

This study used data from the Health and Retirement Study (HRS) data to investigate how an understudied group of Indigenous Older Adults (IOAs) in the United States fared over a 14-year period (2006-2020) in the domain of global cognitive function. The number of IOAs, defined here as Native American and Alaska Natives, will more than double in the next 30 years. Concurrently, the number of IOAs living with cognitive impairments will also increase. Guided by the Minority Stress and Cognition Model, we tested the hypothesis that discriminatory stress increases the risk of cognitive impairment in later life. Using a robust set of psychosocial (e.g. educational quality, perceived everyday discrimination experiences), behavioral (e.g. substance use, exercise), and physiological (e.g. diabetes, hypertension, obesity) risk factors, we modeled the cognition trajectories 186 IOAs using mixed growth curves. We found that one third of these IOAs reported experiencing everyday discrimination at least once per month, the highest of any ethnic group. They also reported high rates of other risk factors for cognitive impairment like low education, SES, and physical activity, and high rates of depression and chronic health conditions. Our analysis found that everyday discrimination was negatively associated with total cognition among IOAs but that this relationship was mediated by allostatic loads. On average, the total cognition scores of IOAs declined significantly faster than those of Whites. This study has important implications for the integration of stress as a mechanism for cognitive decline and the health equity of Indigenous older adults.

